# Indocyanine green fluorescence-navigated laparoscopic metastasectomy for peritoneal metastasis of hepatocellular carcinoma: a case report

**DOI:** 10.1186/s40792-018-0537-x

**Published:** 2018-11-07

**Authors:** Yoshihiro Miyazaki, Masanao Kurata, Yukio Oshiro, Osamu Shimomura, Kazuhiro Takahashi, Tatsuya Oda, Nobuhiro Ohkohchi

**Affiliations:** Department of Gastrointestinal and Hepato-Biliary-Pancreatic Surgery, Faculty of Medicine, University of Tsukuba, 1-1-1, Tennodai, Tsukuba, Ibaraki 305-8575 Japan

**Keywords:** Fluorescence, Hepatocellular carcinoma, Indocyanine green, Metastasis

## Abstract

**Background:**

Indocyanine green (ICG) can selectively accumulate in primary hepatocellular carcinoma (HCC) and its extrahepatic metastases. ICG fluorescence imaging is an extremely sensitive intraoperative tool for detecting HCC foci and can be used to detect impalpable tumors in laparoscopic surgery. Here, we report a case of a 75-year-old man who underwent peritoneal metastasis resection of HCC using a laparoscopic near-infrared imaging system and ICG fluorescence-navigated surgery.

**Case presentation:**

A 75-year-old man was referred to our department for peritoneal metastasis resection of HCC. Two years before admission, he had undergone transarterial embolization and segmentectomy of segment 6 with open surgery for ruptured HCC. Computed tomography revealed a 12-mm peritoneal metastatic lesion on the abdominal wall near the cut surface of the liver. No other metastases were observed; resection of the solitary metastasis was scheduled. ICG (0.5 mg/kg body weight) was intravenously injected, 72 h preoperatively. An endoscopic, ICG near-infrared fluorescence imaging system revealed clear green fluorescence, indicating peritoneal metastasis of HCC on the abdominal wall. The tumor was resected with adequate surgical margin by partially resecting the liver and diaphragm, followed by histological confirmation as peritoneal metastasis of HCC. No recurrence was observed after 12 months of follow-up.

**Conclusions:**

ICG fluorescence can be useful in laparoscopic surgery for identifying peritoneal metastasis.

## Background

Indocyanine green (ICG) is used not only for evaluation of liver function but also for surgical navigations as it emits fluorescence upon exposure to near-infrared illumination [1]. Selective uptake and retention of ICG by hepatocellular carcinoma (HCC) tumors have recently been revealed, and ICG fluorescence imaging has begun to be applied as an extremely sensitive intraoperative tool for detecting foci of HCC [2, 3]. Recent reports have demonstrated that ICG can selectively accumulate in primary HCC and extrahepatic metastases of HCC; additionally, ICG fluorescence imaging presented clear boundaries between tumors and normal tissue [2–5]. Here, we report, to the best of our knowledge, the first case of laparoscopic resection of peritoneal metastasis using ICG fluorescence imaging.

## Case presentation

**Fig. 1:**
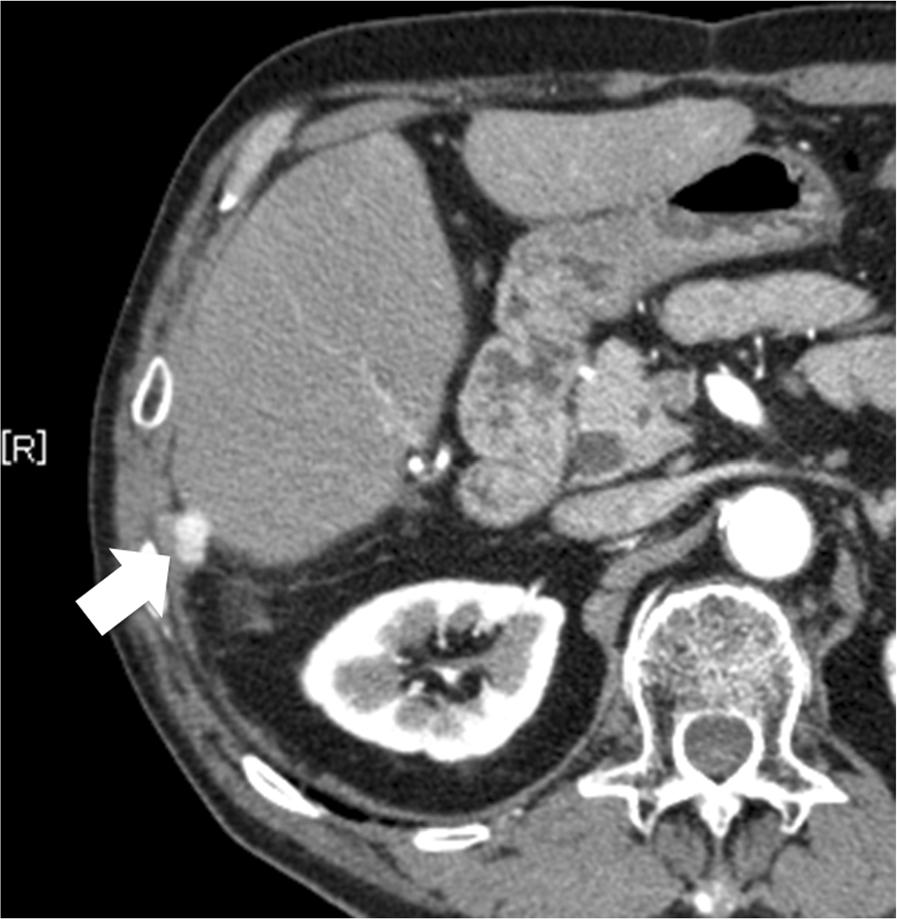
Contrast-enhanced computed tomography findings. Contrast-enhanced computed tomography revealed a peritoneal metastatic lesion of 12 mm in diameter on the abdominal wall near the cut surface of the liver, with enhancement in the arterial phase (arrow)

**Fig. 2:**
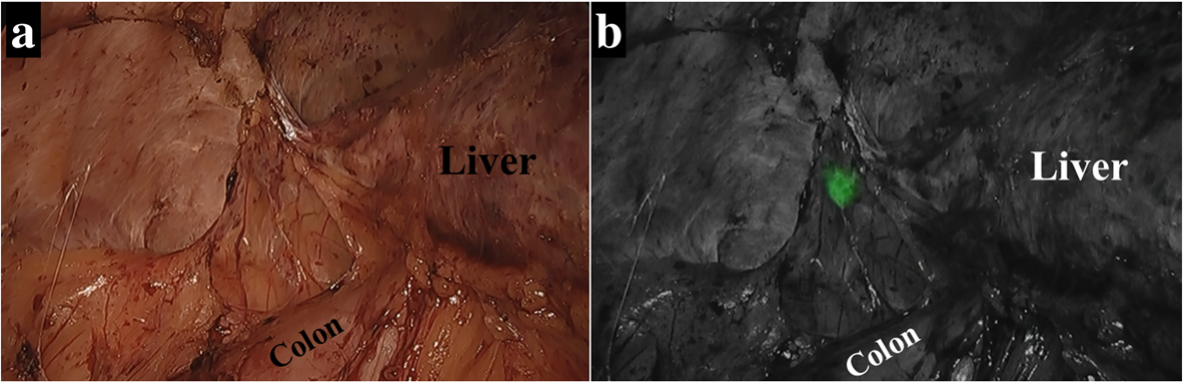
**a** Laparoscopic view after dissection of the adhesion. The location of the tumor was unclear. **b** Endoscopic, indocyanine green (ICG) near-infrared fluorescence imaging using the 1588 AIM camera system. ICG fluorescence mode revealed clear green fluorescence at the tumor site

**Fig. 3:**
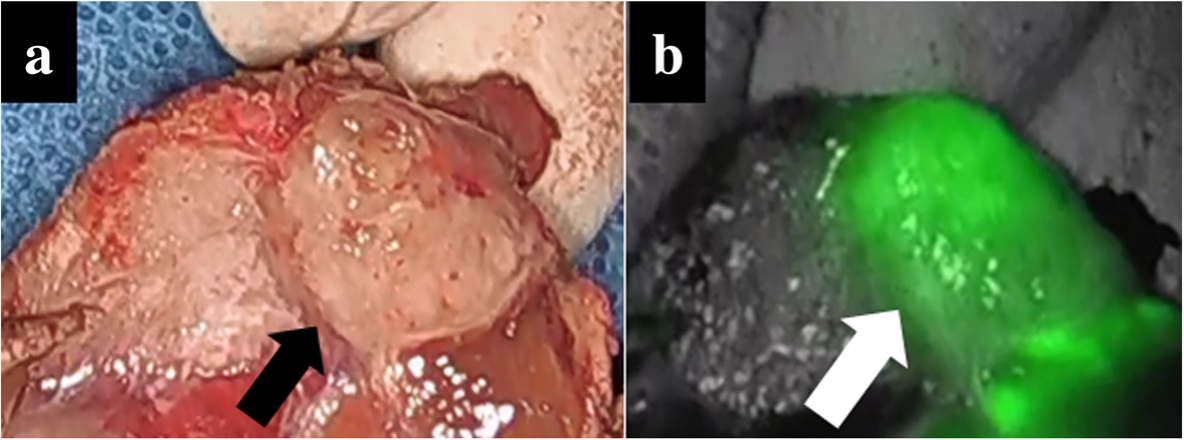
**a** Cut surface of the surgical specimen. Resected specimen contained a 13-mm yellowish tumor (arrow) along the liver and diaphragm. **b** The presence of fluorescence in the tumor was confirmed with illumination using the ICG camera system

A 75-year-old man was referred to our department for resection for peritoneal metastasis of HCC. Two years before admission, he had undergone transarterial embolization and segmentectomy of segment 6 with open surgery for ruptured HCC. Histologically, the tumor was confirmed as moderately differentiated hepatocellular carcinoma. Follow-up computed tomography (CT) revealed a 12-mm peritoneal metastatic lesion on the abdominal wall near the cut surface of the liver (Fig. 1). He had no history of alcohol abuse, hepatitis B or C infection. His liver function was Child-Pugh A, and ICG retention rate at 15 min was 25.2% (normal range; < 10%). Serum α-fetoprotein level and protein induced by vitamin K absence or antagonist-II level were 6.8 ng/mL (normal range; < 10 ng/mL) and 64 mAU/mL (normal range; < 40 mAU/mL), respectively. Contrast-enhanced CT and magnetic resonance imaging revealed that there were no other metastases, and resection of the solitary metastasis was scheduled. ICG was intravenously injected at a dose of 0.5 mg/kg as a routine measure for the evaluation of liver function, 72 h preoperatively. After dissection of the hard and wide range of adhesions, the abdominal cavity was explored with an endoscopic, ICG near-infrared fluorescence (NIF) imaging system (1588 AIM camera system; Stryker Corporation, Kalamazoo, MI, USA) (Fig. 2a). ICG fluorescence mode revealed clear green fluorescence at the tumor site (Fig. 2b). The tumor was resected with adequate surgical margin by partial resection of the liver and diaphragm. Immediately after resection, the surgical specimen was sliced in a plane including the lesion, and the presence of fluorescence was confirmed with illumination using the ICG camera system (Fig. 3a, b). The tumor was histologically confirmed as a peritoneal metastasis of HCC, and the surgical margins were negative. To date, no recurrence has been observed after 12 months of follow-up.

## Discussion

To the best of our knowledge, our case is the first to report the use of ICG fluorescence-navigated laparoscopic metastasectomy in a patient with peritoneal metastasis of HCC. This method is advantageous in facilitating the intraoperative identification of metastatic lesions, particularly during laparoscopic surgery in which the tumor is not palpable.

The survival benefit of removing extrahepatic metastatic lesions of HCC is a clinical question that has not yet been clarified. Long-term survivors in selected cases have been reported in previous literatures [6, 7]. In our institution, the surgical indications for extrahepatic metastases of HCC included all countable lesions that are macroscopically resectable, with intrahepatic lesions that are resectable or controllable by other treatment modalities. In this case, there were no other metastases, and resection of the solitary metastasis was performed.

Recent reports have demonstrated that ICG can selectively accumulate not only in primary HCC but also extrahepatic metastases [2–5]. ICG is selectively accumulated by the hepatocytes and excreted in the bile via an active transport system. However, owing to canalicular transporter anomalies, ICG is captured by HCC cells but cannot be excreted correctly in the biliary canalicules, and thus, ICG accumulates in HCC cells [8]. Ishizawa et al. reported that the fluorescent patterns were classified into three types: total, partial, and rim fluorescent types; these were correlated with tumor differentiation [3]. ICG can accumulate in peritoneal metastases because of their histological similarity to the original HCC. Therefore, ICG fluorescence imaging confirmed the lesion as a peritoneal metastasis. A clinical issue pertaining to this method is the optimal interval between ICG administration and surgery. For detecting an intrahepatic lesion, an interval of at least 2 days is recommended; however, to detect extrahepatic HCC metastasis, the contrast against the liver parenchyma is not essential; the recommended interval is 1–5 days.

ICG-NIF imaging system is advantageous in laparoscopic surgery in which the tumor is not palpable. Laparoscopic surgery is becoming increasingly widespread due to technological developments and improvements in endoscopic technique. However, one disadvantage of laparoscopic surgery is that it is difficult to confirm the tumor and resection margins, owing to the inability to palpate the tumor. Intraoperative ultrasonography is generally useful for detecting liver tumors during laparoscopic hepatectomy. However, regarding small disseminated tumors on the peritoneum, ultrasonography is not useful. The benefit of this ICG fluorescence method is that small metastatic lesions can be easily identified during laparoscopic surgery. Reportedly, IGC fluorescence imaging system can detect 3-mm HCC metastatic lesions [2, 4]. The sensitivity and positive predictive value of intraoperative ICG fluorescent imaging were reported to be 92% and 100%, respectively [4]. However, a limitation of this method is that it cannot detect fluorescence when a lesion is deeply located in a tissue. Thus, tumors at a depth of > 5–10 mm from the surface could not be identified [2–4]. In this case, the tumor location could be visualized by the ICG-NIF imaging system, and we could detect and resect the metastatic tumor on the peritoneum. Without the ICG-NIF imaging system, the peritoneal tumor could not be detected because the tumor was not visible in the surrounding hard adhesion and adipose tissue.

## Conclusions

In conclusion, we presented a case of laparoscopic resection of peritoneal metastasis using ICG fluorescence imaging. This method is useful for the detection and safe resection of peritoneal tumors during laparoscopic surgery.

## Abbreviations

CT: Computed tomography
HCC: Hepatocellular carcinoma
ICG: Indocyanine green
NIF: Near-infrared fluorescence
